# Antipsychotic Medication Review of Care Homes Residents in Neath Port Talbot (NPT)

**DOI:** 10.1192/bjo.2025.10539

**Published:** 2025-06-20

**Authors:** Nermeen Ahmed

**Affiliations:** Swansea Bay University Health Board, Swansea, United Kingdom

## Abstract

**Aims:** To reduce or stop inappropriate prescriptions of antipsychotic medication in Older Adults with dementia or functional illness residing in care homes in NPT, by ensuring adequate and timely reviews of antipsychotic medications.

It also compares its findings with the last audit results in October 2022.

**Methods:** Retrospective Audit included patients in care homes under CHIRT from NPT, a total of 164 patient were on antipsychotic medication starting this audit compared with 146 total number of patients on last audit in 2022.

Audit period: 10/5/2023 to 10/05/2024.

Data were collected from the antipsychotic register, reviewing the initiation and monitoring charts to assess patients for side effects.

Patients were classified according to Age, Gender, Diagnosis, Prescribed Antipsychotic and status of the antipsychotic reviews.

**Results:** A larger number of patients on antipsychotics compared with previous audit with expected demographics and side effects given the offered medication.

A total of 83 patients were continued on antipsychotics, 56 patients discontinued antipsychotics, with 25 reported deaths within the audit year. This shows a significant increase in number of patients discontinuing antipsychotic medication by 34% of total numbers in comparison to the 2022 audit.

Antipsychotic review status was up to date for 68 patients, overdue for 12 patients and not stated for 4 patients. This shows a significant decrease in number of patients with overdue reviews for antipsychotic medication currently at 19% of total reviews in comparison to last audit’s results of 61% of reviews.

**Conclusion:** It is good practice to review initiation of antipsychotics regularly once in 6 weeks–3 months in accordance with NICE Guidelines.

It is good practice to monitor antipsychotics in care homes once in 6 months to follow NICE Guidelines and ensure regular reviews.

It is recommended to audit prescribing of antipsychotic medication once in 6 months to maintain good medical practice.

